# Midwifery

**Published:** 1837-10

**Authors:** 


### MIDWIFERY.

*On the Puerperal Fever which prevailed in the Lying-in Hospital at Kiel, from September,* 1834, *to March,* 1835, *and during the Winter of* 1835-6; *and on the Treatment by Ice.* By Dr. Michaelis.

Until the year 1834, this institution had been almost entirely free from puerperal fever, at least in a malignant form. The situation of the building is very far from being favorable, nor does the arrangement of the wards, &c. appear to be much better. The chief cause of the complaint must be considered to be of an epidemic nature, because, previous to its appearance in the hospital, Dr. M. had seen several cases in private practice; although otherwise it is not frequently witnessed in the district. Several were fatal cases. It is remarkable that each appearance of the disease in the hospital had been preceded by the death of a patient from difficult labour. This was not only the case in the two last epidemics, but also in 1819 and 1830, when an unusual mortality followed similar fatal cases. The contagious nature of the disease was but too evident, and the rapidity of its action almost incredible. Several women, perfectly healthy, came into the hospital in labour, and were scarcely delivered before the disease burst out in full activity. The contagious nature of it was further proved by its resisting every attempt to arrest its progress, until the whole building had undergone a thorough purification and refurnishing. With regard to the mortality, this varied very much, according to whether the patient was attacked immediately after labour, or not until several days had elapsed. In the first case it was very fatal, in the other never. The ovaries appeared to be the chief seat of the disease, and where the pain was first complained of. Abscess of the ovaries and suppuration of the veins were observed in several cases.

The most constant symptoms were anxiety in breathing, from complete inaction of the abdominal muscles; hence the laborious heaving of the thorax, the extreme rapidity of the pulse, at one moment full, at another small; sleeplessness, and the local symptoms of the affection. Diarrhœa alternated with constipation; occasionally vomiting and tympanitis. The profuse and reeking perspirations, and the early appearance of diarrhœa, were not critical; whereas, diarrhœa at a later period seemed to form a decided crisis, and was often exceedingly profuse. Dr. Michaelis observed, as a remarkable fact, that the features of the patient, till the last moment, underwent but little alteration, except that there was an expression of anxiety; she remained conscious to the last; and in most of the fatal cases there was no appearance of delirium.

The treatment required to be varied. Where the disease appeared after the third day, local depletion with leeches, and poultices, were the chief treatment. Bleeding was seldom had recourse to. The secretion of milk was kept up as much as possible, and small doses of calomel internally, or neutral salts where the bowels were confined. This treatment was always effective and sufficient; but in difficult cases, where the patient was attacked shortly after labour,—viz. the first or second day,—Dr. M. found no mode of treatment of any avail in the first epidemic; not a single patient escaped. Powerful antiphlogistic treatment; calomel in small and then in large doses, smart purging, camphor, turpentine injections, &c. &c. were all equally unsuccessful in producing even the most temporary abatement of the symptoms. It was only by the use of ice that Dr. Michaelis succeeded in saving the last patient who was severely attacked during the first epidemic: it was suggested to him by M. Kirchhoffer, a student, who had seen it used at Copenhagen. Dr. M. used ice both externally and internally. A piece of ice, of about the size of a finger, was given to the patient every half or quarter of an hour, according to her own inclination and the severity of the disease; and this was continued for two or three days. As soon as she began to dislike it, it was stopped. Externally, a very large bullock's bladder, partially filled with pounded ice or snow, was laid upon the abdomen, so that it was covered with a layer of ice an inch in thickness. As soon as the ice was melted, a fresh bladder was applied; and this was continued, according to the patient's feelings, without intermission, for seventy hours. The patients express great relief when this is applied. In some cases the weight of ice was irksome, but this may be relieved by placing some thin cask-hoops across the abdomen beneath the bladder; the weight is by this means more equally diffused. The almost instant effect produced by this application was the feeling expressed by the patient—no remedy has proved so grateful to her feelings as this; and Dr. M. never ventured to employ it without this indication.

The most remarkable change in the symptoms is of the pulse. In the course of a few hours it sinks ten, or even thirty, beats in frequency, and becomes more distinct and free. When this effect was not produced, the case generally soon proved fatal. Other changes followed at a later period,—viz. rest, and sleep for several hours, (sometimes, it is true, the symptoms will return again more violently after it, but still sleep is a most favorable symptom;) cessation of vomiting; return of peristaltic action; after a time the bile again appears in the evacuations. The distended state of the abdomen diminishes, partly from the astringent effects of cold, and partly from discharge of flatus. A profuse watery diarrhœa soon comes on, which appears to be the first effort towards convalescence: this occasionally proceeds to a great extent, but is nevertheless very beneficial. Dr. M. thinks that the powerful application of ice is capable of producing absorption of those immense effusions which take place in the abdominal cavity. In some cases he observed violent pains in the shoulders after the application of the ice, and at a later period, during the patient's recovery, a variety of rheumatic and catarrhal affections; but they were of no serious importance. Dr. M. used no internal remedies in conjunction with the ice. Leeches applied upon the most painful spot, and free venesection, were now of service; whereas this latter, when used at an earlier period, seemed only to accelerate the fatal termination.

The two following cases we select as a specimen of this treatment and of its results, not because they were successful, (for Dr. M. has recorded some fatal cases where ice was employed.) We have selected these two because, from the peculiar circumstances of the case, the exhibition of ice was tried more fairly than in some of the unsuccessful ones.

Case i. A robust primipara was delivered on the 12th of January, 1836, after an easy and natural labour. She had an early and abundant supply of milk. On the 14th, (third day after delivery,) she was seized with violent pain in the abdomen, preceded by a rigor, and the uterus became very tender to the touch. Twenty leeches and the internal exhibition of ice-pills (*eispillen*,*) relieved these symptoms in twenty-four hours.—Jan. 19. Had another rigor and a return of the abdominal symptoms. Pulse 120; great depression of spirits, although otherwise of a remarkably cheerful disposition. Twenty leeches were ordered, and ice-pills. —Jan. 20, (second day of the attack.) Much the same. Still plenty of milk. Bowels opened six times. Repeat the leeches and ice-pills.—Jan. 21, (third day.) Innumerable evacuations; pain less, milk diminished; pulse 100. Continue the

* Quere: were these little pieces of ice swallowed, or allowed to melt in the mouth? We must presume the former.—Rev.

ice.—Jan. 22, (fourth day.) Feels well, except that there is a small spot on the left side of the uterus, which is still painful. Omit the ice.—Jan. 23, (fifth day.) Convalescent; the diarrhœa continues nevertheless. The milk has disappeared, the child having died from aphthæ.—The diarrhœa continued profusely until the 29th of January; it then ceased of itself, and the patient's appearance was very good for some days. In the beginning of the following month, she was attacked with inflammation in the veins of the left leg; the veins being hard and excessively painful along the whole limb, but with little fever. Friction with unguent. hydr. ciner. was used until a slight degree of salivation was produced; the symptoms abated, and she left the hospital in good health on the 14th of February.

CASE II. A young woman, æt. 20, in her first pregnancy, who had been under medical treatment (viz. was bled,) a fortnight before on account of bronchitis, was delivered of a healthy child, after a labour of five hours' duration, on the morning of Jan. 27, 1836. The pains were weak, and, although she had recovered from her attack, yet, as she appeared still delicate, she was delivered in bed. The placenta quickly followed the birth of the child, and she felt perfectly well.

Jan. 28, (first day of the attack.) No sleep during the night. After a shivering fit, she was seized with heat, quick breathing, much anxiety, violent thirst, and vomited twice. In the morning, the pulse was 112, full; the abdomen exquisitely tender. Sixteen leeches were applied to the abdomen, followed by poultices; cold water for her drink. Towards evening the symptoms were worse, except a very transient alleviation of pain by the leeches. Iced water; and, at seven o'clock P.M., ice was ordered to be taken every half-hour, and an enema. The pulse had risen to 120.

Jan. 29, (second day.) Very restless during the night; violent pain; three copious feculent evacuations. Towards morning, she slept for two or three hours. At noon, the pulse was 140, full, and hard; determination to the head; the abdomen intensely painful, and already tympanitic; bitter eructations, followed by vomiting of a bitter watery fluid. Let her have ice every quarter of an hour, and be bled to twenty ounces. Towards evening, sixteen leeches were applied to the epigastrium, which had become painful; after which she slept for two hours, during which the pulse fell to 116, soft, and full, with general perspiration.

Jan. 30, (third day.) Has had a bad night. Vomited four times, during which she threw up two worms alive. Bowels open twice. Violent burning pain in the abdomen; vertigo; painful eructation; hiccup; abdomen immensely distended; tympanitic. Pulse 132, full and hard; reeking perspiration; feels that she is dying; much depression of mind. V.S. ad deliquium, (℥xvj.) Dislikes the ice, which is therefore stopped. At noon, pulse 132, soft; much anxiety. A large bladder, with snow, was placed over the whole abdomen, (from the scrobiculus cordis to the pubes,) and repeated every half-hour. The application feels very grateful. At her earnest request, she was allowed iced beer for her drink. The favorable effects of this treatment soon manifested themselves: the painful eructations and hiccup ceased, and she had a copious evacuation of whitish grey fæces. Calm sleep for several hours followed; the breathing became deep and quiet; the pulse fell to 108, full and soft; sense of burning diminished.

Jan. 31, (fourth day.) Her night was good until three o'clock, since which she has been restless, has vomited three times, throwing up a quantity of dark-green bile and two more worms. Has passed involuntarily several grey fluid stools. The abdomen was nevertheless not so painful at its lower part, although more distended: on percussion here, it had entirely lost the intestinal sound, *and fluctuation could be distinctly felt.* It was evident that fluid was already extravasated into the abdominal cavity, confirmed by the temporary remission of the symptoms. Much tenderness above the umbilicus; pulse 120, small, hard, compressed. The day followed without any material change. She vomited once a quantity of watery fluid, and another worm. Several watery grey-coloured stools were passed involuntarily. Hitherto, during the whole course of the attack, the child had been regularly applied to the breast, and appeared to obtain milk, although the breast was somewhat flaccid. The lochia had entirely ceased. The expression of her countenance was still tolerably good.

Feb. 1, (fifth day.) She passed a good night until four o'clock, dozing. Very frequent evacuations, for the first time of a yellow colour, showing that the peristaltic motion had returned. Violent stitch in both shoulders. Abdomen somewhat less; fluctuation disappeared; epigastrium less painful. It was attempted to leave off the application of ice to the abdomen, having been persevered in for forty-four hours; but she had scarcely passed an hour before the burning sensation in the abdomen returned, with painful eructations and anxiety; the ice-bladders were therefore again applied, and repeated every hour. The day passed favorably. She dozed a good deal, passed some flatus and innumerable involuntary evacuations. Pulse 120: in the morning it was small, hard, and compressed; in the evening soft, somewhat empty, but less compressed. Features sunken. She expressed a wish for lemon-juice and water as a drink.

Feb. 2, (sixth day.) Passed a good night until three o'clock; after which was restless, and complained of pain in the right hypochondrium, which is distended and hard. Innumerable evacuations passed involuntarily; has an expression of suffering; pulse 120, hard, small, and compressed. At noon she wished to leave off the applications of ice, which had been continued, with a very short interval, for three days. Eructation followed, and the pulse rose to 128; the evacuations contain dark green bile.

Feb. 3, (seventh day.) The evacuations ceased during the night; this was followed by restlessness and sensation of burning. Ice was therefore again applied twice, and this was followed by green evacuations, and quiet. In the morning she felt tolerably well; her spirits have returned. The abdomen is almost entirely without pain on pressure, and but slightly distended. Pulse 120, hard; otherwise good. She expressed a wish for stewed prunes *(Pflaumen suppe,)* with lemon juice, which she took with relish. Since yesterday the breasts have become somewhat larger; the lactiferous tubes may be distinctly felt. In the evening, after putting on clean linen, she felt comfortable. Her only complaint is of hunger. Has passed the first voluntary evacuations for the last four days; they are still frequent, but less copious.

Feb. 4, (eighth day.) Feels well. Evacuations, although frequent, are now again feculent; pulse 110. At nine in the evening, the burning sensation in the abdomen suddenly returned, with anxious breathing, during which the thorax alone acts, the abdomen and diaphragm being perfectly motionless. Sensation of suffocation; pulse 128, hard, and suppressed; the look is vacant, with slight strabismus. At half-past ten a bladder was applied to the abdomen, which immediately relieved every symptom.

Feb. 5 to 9, (ninth to thirteenth day.) On the whole she continues to improve. Sharp pains in the abdomen continued to appear occasionally; for which fomentations of brandy, and afterwards washing with cold vinegar and water, seemed useful. Hitherto the bowels had been opened more than six times every day; when this was not the case, she did not feel so well. The fæces and urine generally pass involuntarily, and sometimes unconsciously. The urine now resembles milk, slightly tinged with blood. Although the tongue is brown and dry, her appetite is tolerably good, and she has taken some bread with the prunes. Pulse still 110. Although the nipples are very sore, she continues to suckle her child. Has taken no medicine, as nature was evidently capable of effecting the process of absorption. The condition of the abdomen, as also her former symptoms, leave no doubt as to the existence of extensive effusions of coagulable lymph. At a later period these could be felt distinctly.

Feb. 10 to 16, (fourteenth to twentieth day.) The milk has disappeared; she improves slowly; her appetite has not yet returned. Excretions, for the most part, are natural; two or three evacuations daily, increased in quantity. Occasional inability to pass water; slight cough.

March 10. Was discharged. Still weak, and with distinct indurations here and there in the abdomen. This was carefully examined about the end of February, when adhesions of the bowels were distinctly traced, under the form of hard compact tumours. These filled the whole abdominal cavity below the anterior and superior spinous process of the ilium; an indurated mass, as large as a fist, could also be felt beneath the right lobe of the liver. Towards the end of the month she suffered frequently from vomiting, diarrhœa, and want of appetite; the urine became as thick as milk, and during this state the indurations became much softer; that of the liver alone could be felt when she left the hospital. As these attacks of vomiting seemed evidently a part of the sanitory process of nature, especially as the pulse always became more quiet, no medicine was given to stop it: the abdomen was well covered with flannel, and frictions used with volatile liniment. *Neue Zeitschrift für Geburtshülfe.* Vol. iv. No. 3. 1836.

---

*Case of Cæsarean Section, performed with success for the fourth time on the same Individual.* By Dr. MICHAELIS, of Kiel.

AN account of the three preceding operations, and of the case generally, is given in our second volume, p. 270. The first operation was performed in June, 1826, the woman being then in her twenty-ninth year; the second in January, 1830; the third in March, 1832. This woman became once more pregnant, and, the operation being equally necessary as before, it was performed by Dr. Michaelis, on the 27th June, 1836, after the patient had been in labour three days. The new incision intersected the second and third cicatrices, and the uterus had become so completely adherent to the abdominal parietes that the peritoneal cavity was not laid open. On the third day after the operation, the patient was threatened with alarming symptoms of peritonitis, accompanied by tympanitis, which speedily yielded to the internal exhibition of ice and a few doses of calomel. The external wound could not heal, on account of the gaping of the uterine opening, which kept apart the adherent margins of the divided skin, and thus converted the wound of both integument and uterus into a single symmetrical aperture. On the 1st of August, (the period at which the last report is dated,) the uterine aperture was rather more than half an inch in extent; and this diminution appeared to be solely dependent on the gradual contraction of the uterus, inasmuch as the healing process itself was not then contemplated. Nevertheless, the patient left her bed daily, and her general health was good. She herself suckled her child, which was thriving well.

[An interesting point connected with this case is the occurrence of peritonitis after the fourth operation, in which instance alone, it will be remarked, the serous sac was *not* opened, and was therefore unexposed to the influence of external agents, as the atmospheric air, &c.

A medical friend suggests the expediency, in cases of hopeless deformity of the pelvis, that the fallopian tubes should be divided during the Cæsarean operation: in the event of a successful result to the operation, this proceeding would, of course, do away with all risk of a second.]

*Pfaff's Mittheilungen.* Heft vii.-viii. 1836.

